# Protective effect of sevoflurane preconditioning on ischemia-reperfusion injury in patients undergoing reconstructive plastic surgery with microsurgical flap, a randomized controlled trial

**DOI:** 10.1186/s12871-016-0230-1

**Published:** 2016-08-22

**Authors:** Claudia Claroni, Giulia Torregiani, Marco Covotta, Maria Sofra, Alessandra Scotto Di Uccio, Maria E. Marcelli, Alessia Naccarato, Ester Forastiere

**Affiliations:** 1 Department of Anaesthesiology, Regina Elena National Cancer Institute, V. Elio Chianesi 53, 00144 Rome, Italy; 2 School of medicine, University Hospital Center Tor Vergata, V.le Oxford 81, 00133 Rome, Italy

**Keywords:** Free flap, Ischemia reperfusion injury, Near infrared spettroscopy, Preconditioning, Sevoflurane

## Abstract

**Background:**

In many clinical conditions that involve free flaps and tissue transplantations the possibility of minimizing ischemia-reperfusion injury can be a determinant factor for the success of the surgery itself. We hypothesize that preconditioning with sevoflurane is a protective factor against ischemia-reperfusion injury.

**Methods:**

In this randomized controlled trial, patients ASA I-II undergoing breast reconstruction with deep inferior epigastric perforator flaps were allocated into two groups and analyzed: group BAL included patients who received balanced anesthesia with sevoflurane for 30 min before removal of the flap and throughout the surgery. The TCI group included patients who received a total intravenous anesthesia with propofol and remifentanil. We evaluated regional tissue oximetry at the end of the surgery and at 4, 12 and 20 h after surgery. Other assessed parameters were: blood lactate clearance, alanine aminotransferase, aspartate aminotransferase, lactic dehydrogenase, creatine phosphokinase.

**Results:**

In total 54 patients, twenty-seven per group, were analyzed. There was a significant increase of the average value of regional tissue oximetry measured 4 h after surgery in the BAL group compared to the TCI group: BAL: 84.05 % (8.96 SD); TCI : 76.17 % (12.92 SD) (*P* = 0.03), but not at the other time frames. The creatine phosphokinase value was significantly lower in the BAL group at the end of surgery, but not at the other time-frames. There were no significant differences in blood levels of other markers.

**Conclusions:**

From our results, the positive preconditioning impact of sevoflurane on ischemia-reperfusion injury in patients undergoing free flap surgery is expressed in the early postoperative hours, but it does not persist in the long-term.

**Trial registration:**

ClinicalTrial.gov identifier: NCT01905501. Registered July 18, 2013

## Background

The ischemia-reperfusion (IR) injury is a pathological condition characterized by an initial restriction of blood flow to an organ, followed by restoration of perfusion and oxygenation**;** this generates a set of complex mechanisms that culminate with vascular, biochemical and cellular changes. The ischemic period produces endothelial stress, cellular alteration and reduced levels of high-energy metabolites such as adenosine triphosphate (ATP), along with the production of free radicals and neutrophilic activation. Reperfusion provides a significant amount of oxygen that is converted into reactive species of oxygen, which elicit changes in mitochondrial permeability, alterations of the membranes and the emission of preapoptotic molecules [1]. Free radical activation of neutrophils amplifies endothelial damage; the modification of intracellular ions’ concentration, with ATP depletion, contributes to the processes of cell necrosis [2].

IR injury is inevitable in many clinical conditions that involve tissue transplantation and free flaps. In reconstructive plastic surgery with free flaps, the control of IR injury can have a significant impact, playing a decisive role in the outcome of the surgery itself. For these reasons, research on methods that protect tissues from damage associated with molecular IR is particularly important. In recent years, the role of sevoflurane administration before the occurrence of the ischemic process (preconditioning) has been widely investigated as a protective factor against IR injury in different settings [3].

Many studies have been conducted on sevoflurane myocardial preconditioning in cardiac patients undergoing cardiac [4] and non-cardiac surgery [3–5], and a recent meta-analysis showed that the use of halogenated anesthetics in cardiac surgery reduces the rate of morbidity and mortality [6].

In general, the effect of cardiac sevoflurane preconditioning has been related to the endothelial protection [7] and to the beneficial effects on inflammatory response [8, 9]. Therefore, it can reasonably be assumed that this method has a protective effect on other ischemic tissues as well [10, 11].

Our study aims to evaluate the possible protective effect of a balanced anesthetic technique (BAL), compared to a total intravenous anesthetic technique using target-controlled infusion (TCI), on ischemic reperfusion conditions, inevitably generated in patients undergoing reconstructive surgery with a microvascular flap. For this purpose, we assessed the metabolic status of the flaps by using continuous regional tissue oximetry (rSO2) and by monitoring the levels of biochemical markers in the postoperative period.

At the current state of our knowledge, there are no other studies evaluating the effect of sevoflurane preconditioning on microvascular free flaps in plastic surgery.

Our hypothesis is that in the group of patients receiving sevoflurane preconditioning there is a statistically significant increase in rSO2 measured by employing a near-infrared spectroscopy monitor.

## Methods

A multi-center prospective, randomized study was conducted in the period between July 2013 and November 2014, at the Regina Elena National Cancer Institute, Rome (Italy) and at the Sacro Cuore Catholic University, Rome (Italy). The study was approved by the Central Ethics Committee of National Cancer Institute Regina Elena and Sacro Cuore Catholic University, Rome, Italy (Chairperson Dr Anna D’Ambrosio), with Protocol n. CE/67/13, and retrospectively registered with ClinicalTrial.gov identifier: NCT01905501. Clinical investigation was conducted according to the principles expressed in the Declaration of Helsinki.

After written informed consent, ASA I-II patients candidate for free deep inferior epigastric perforator (DIEP) flaps for breast reconstruction were enrolled. Patients under the age of 18, those with known unusual reaction to anesthetic drugs, the ones with a history of vascular disease or of bleeding diathesis and others with nicotine addiction were all excluded from the study.

Patients were randomly divided into two treatment groups by an operator who is not directly involved in the study and according to a specific dedicated software, developed in-house, by GW Basic (Microsoft Corporation, USA) programmer, which generated an assignment code verified immediately before inducing anesthesia. Surgeons were blinded to the intervention and a blinded observer recorded the outcome.

In both groups, patients were premedicated with midazolam 0.01 mg/kg and general anaesthesia was induced with fentanyl 3–5 mcg/kg, propofol 2 mg/kg, and cisatracurium 0.07 mg/kg. After tracheal intubation, in the first group (BAL) anesthesia was maintained with a mixture of sevoflurane/oxygen/air, adjusted to provide an end-tidal sevoflurane of 1.5–2 vol.%, and remifentanil, adapted according to a TCI range of 2–4 ng/ml. This anesthesia was always started at least 30 min before flap removal. In the second group (TCI) anesthesia was maintained with propofol 3–4 μg/ml and remifentanil 2–4 ng/ml according to TCI protocol. In both groups, patients were ventilated with positive pressure ventilation in volumetric mode. Ventilator parameters were adjusted to perpetuate an end-tidal CO2 (etCO2) between 30 and 37 mmHg and a tidal volume of 6–8 ml/kg.

The same fluid protocol was used by the anesthesiological equipe in both groups: the fluid therapy regime was mainly restrictive, according to basal infusion of crystalloid variable from 2 to 4 ml/kg/h. Arterial blood pressure (ABP) was regulated by titraing remifentanil and fluid administration in order to maintain a target at values between 65 and 95 mmHg. It was possible to administer boluses of colloids (HES 130/0.4) of 250 ml in 15 min if mean arterial pressure was ≤ 70 % of preinduction.

The standard monitoring for all patients consisted in continuous electrocardiogram, heart rate (HR), ABP measurement, pulse oximetry (SpO2), inspired and expired gas and capnometry. In all patients, regional tissue oximetry (rSO2) was measured employing a near-infrared spectroscopy (NIRS) monitor with In-Vivo Optical Spectroscopy System (INVOS™, Covidien, Boulder, CO). The INVOS™ sensors were placed directly on the transplanted flap at the end of surgery. The spectrophotometric values were determined at the end of the surgery (T0), and at 4, 12 and 20 h after surgery (respectively T1, T2 and T3). Postoperatively, ABP, HR, SatO2 were recorded at the same time point. Other parameters evaluated were: blood lactate clearance, alanine aminotransferase (ALT), aspartate aminotransferase (AST), lactic dehydrogenase (LDH), creatine phosphokinase (CPK). All blood markers were quantified preoperatively, at the end of the surgery, and at 4 and 20 h after surgery.

In all patients the main hemodynamic parameters (ABP, HR, SpO2, FiO2, etCO2) were recorded every hour during surgery.

Furthermore, duration of surgery, incidence of patients who were transfused, incidence of patients with surgical site infection, as stated by the Centers for Disease Control (CDC), and those in need of surgical re-exploration within 7 postoperative days were also assessed.

### Statistical analysis

The study was designed as a two-arm parallel, prospective, randomized trial. The primary endpoint was considered the rSO2. Assuming an alpha error of 5 %, power of 80 % and a dropout rate of 10 % and using a previous data analysis of a group of patients that preceded this study and which was not included in it, we considered rSO2 = 80 % ± 10 (sd) as a reference value for the control group and we theorized a 10 % improvement. We estimated a sample size of 54 patients (27 per group) to detect a minimum 10 % (effect size d = 0.8) estimated clinically significant difference between the groups.

Two-way repeated-measures analysis of variance (ANOVA) was used to test the differences in continuous variables between groups and over time. This analysis allowed us to correctly evaluate repeated observations on patients and to test the effect of this experimental treatment and the variables’ trend throughout the observation time. Differences between continuous variables at specified time intervals were subsequently evaluated with Student’s *t*-test. Dichotomous variables were analyzed using Fisher’s exact test. Variables are presented as means.

The threshold for statistical significance was set at *P* < 0.05. Computerized statistical analysis was performed with the Statistical Package for the Social Sciences software, version 20.0 (IBM SPSS Statistics, Chicago, IL, USA).

## Results

**Fig. 1:**
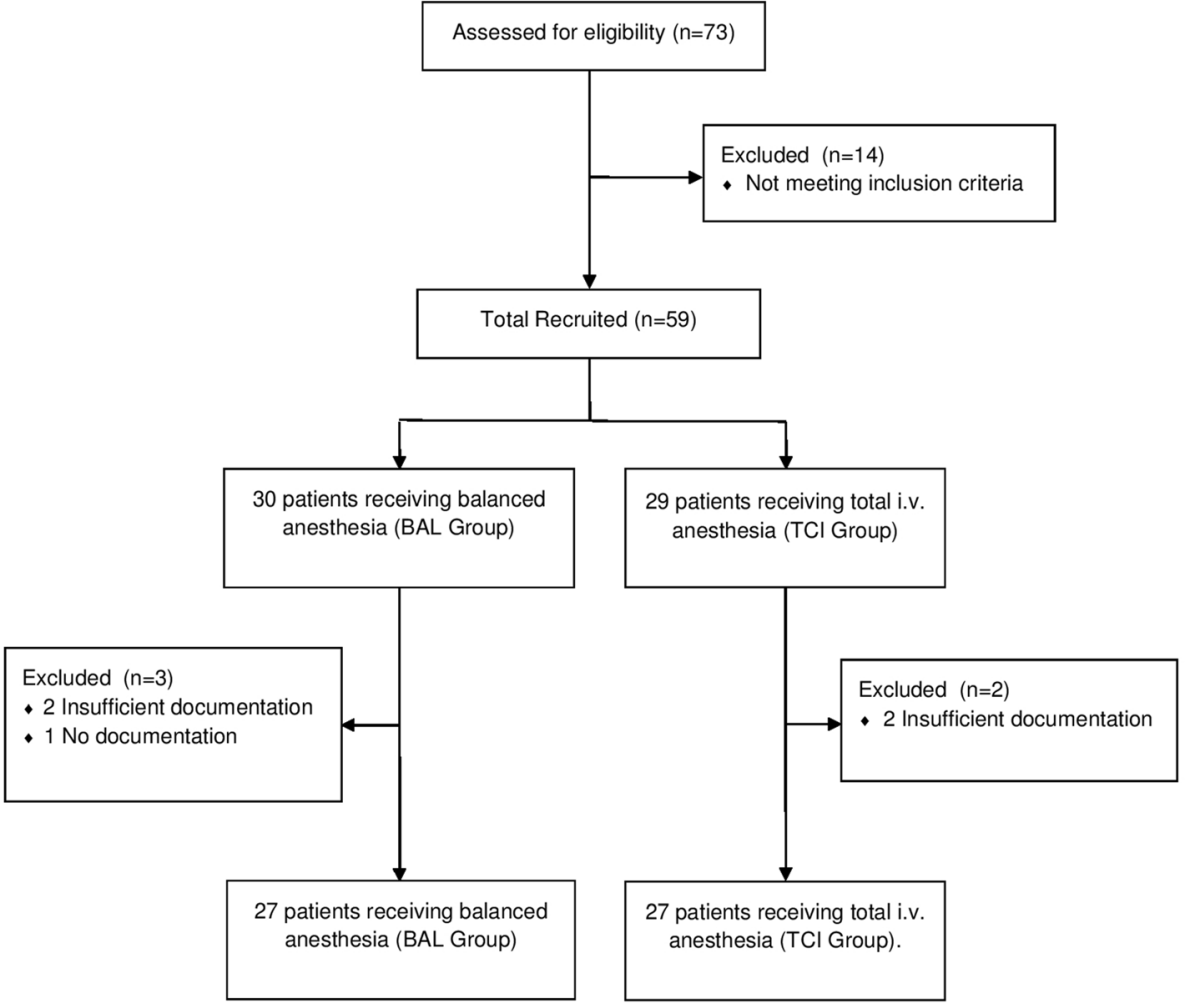
Patient disposition

The flowchart of the patients who participated in the study is shown in Fig. 1. In total, 54 patients were analyzed after randomization: 27 patients were assigned to the BAL group and 27 to the TCI group. The demographic and clinical data is presented in Table 1. There were no significant differences between groups in terms of age, body mass index (BMI), comorbidity, type and duration of surgery, ASA classification and cancer type.

**Table 1:** Demographics, perioperative and postoperative profile (Results, pag 8, line 156 and page 9, line 177)

	BAL Group	TCI Group	*P* value
	(*n* =27)	(*n* =27)	
Age (years)	50 (7.5)	52.7 (7.8)	
BMI (kg m^−2^)	22.2 (2.7)	23.1 (2.9)	
ASA score (I/II)	22 (81.4)/5 (18.6)	25 (92.6)/2 (7.4)	
Comorbidities:			
dyslipidemia	2 (7.4)	0	
hypertension	1 (3.7)	1 (3.7)	
Patients transfused	0	0	
CDC surgical site infection	1 (3.7)	3 (11.1)	0.64
Surgical re-exploration	6 (22.2)	4 (14.8)	0.49
Lenght of surgery (min)	398 (39.1)	417 (50)	0.12

Data is expressed as mean (SD) or number of patients (%)

*BMI* body mass index, *AS*A American Society of Anesthesiologists, *SD* standard deviation, *CDC* Centers for Disease Control

**Fig. 2:**
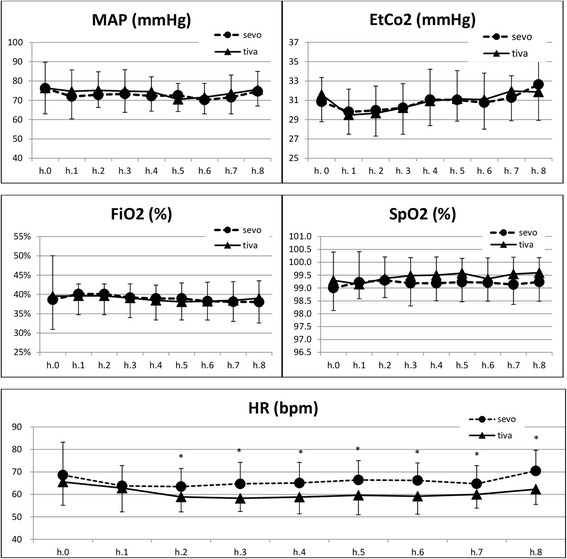
Intraoperative data. All data presented as mean (standard deviation). * Significant. MAP: mean arterial pressure. EtCO2: end tidal carbon dioxide. FiO2: fraction of inspired oxygen. SpO2: saturation of peripheral oxygen. HR: heart rate. Bpm: beats per minute. h.: hour of surgery

The intraoperative hemodynamic data recorded is shown in Fig. 2. In the BAL group, the heart rate remains significantly higher for the entire duration of surgery. There are no significant differences between the groups regarding other intraoperative values.

The mean regional tissue oximetry value in the four time frames for the BAL group and TCI group respectively was as follows: T0: 81.8 % (8.4 SD) and 76.8 % (11.4 SD) (*P* = 0.13); T1: 84 % (8.9 SD) and 76.1 % (12.92 SD) (*P* = 0.03); T2: 84.8 % (10 SD) and 80.3 % (14.4 SD) (*P* = 0.27); T3: 85.4 % (9.9 SD) and 80.6 % (18.6 SD) (*P* = 0.32). Values of blood lactate clearance, alanine aminotransferase, aspartate aminotransferase and lactic dehydrogenase are not statistically different in the two groups for the duration of the monitoring. However the creatine phosphokinase value was significantly lower in the BAL group at the end of the surgery, but not at the other time frames: preoperatively: 68.7 (54 SD) in the BAL group and 56.4 (31.1 SD) in the TCI group (*P* = 0.31); at the end of the surgery: 133.6 (74.1 SD) in the BAL group and 184.1 (100.2 SD) in the TCI group (*P* = 0.04); at 4 h after surgery: 293.8 (238.6 SD) in the BAL group and 294.2 (160.7 SD) in the TCI group (*P* = 0.99); at 20 h after surgery: 495.2 (530.2 SD) in the BAL group and 427.6 (305.2 SD) in the TCI group (*P* = 0.58) (Table 2).

**Table 2:** Main results. (Results, pag 8, line 173)

	BAL Group (*n* = 27)	TCI Group (*n* = 27)	*P* value
rSO_2_ (%)			
end of surgery	81.8 (8.41)	76.77 (11.42)	0.13
4 h after surgery	84.00 (8.96)	76.16 (12.91)	0.03*
12 h after surgery	84.85 (10.04)	80.33 (14.42)	0.27
20 h after surgery	85.40 (9.97)	80.61 (18.66)	0.32
mean	81.75 (11.07)	78.72 (12.89)	0.37
Clearance Lac			
end of surgery/4 h after surgery	−98.18 (89.12)	−83.85 (107.61)	0.60
end of surgery/20 h after surgery	−35.79 (97.64)	−37.26 (52.59)	0.95
CPK (iu/l)			
preoperative	68.69 (53.99)	56.37 (31.09)	0.31
end of surgery	133.61 (74.07)	184.10 (100.26)	0.04*
4 h after surgery	293.81 (238.66)	294.23 (160.7)	0.99
20 h after surgery	495.19 (530.22)	427.60 (305.19)	0.58
LDH (iu/l)			
preoperative	160.11 (93.96)	177.48 (120.56)	0.56
end of surgery	174.70 (120.27)	171.88 (76.49)	0.92
4 h after surgery	389.07 (1182)	188.11 (101)	0.39
20 h after surgery	174.44 (78.67)	184.28 (94.6)	0.68
AST (iu/l)			
preoperative	15.77 (6.76)	22.85 (22.72)	0.12
end of surgery	15.40 (5.79)	37.50 (74.74)	0.13
4 h after surgery	16.22 (6.81)	32.10 (48.63)	0.10
20 h after surgery	19.85 (11.74)	28.65 (31.89)	0.19
ALT (iu/l)			
preoperative	16.85 (15.5)	20.03 (19.71)	0.51
end of surgery	13.56 (7.68)	38.69 (93.04)	0.17
4 h after surgery	14.60 (9.62)	32.60 (60.81)	0.13
20 h after surgery	15.80 (9.73)	27.30 (51.18)	0.25

All data presented as mean (standard deviation). *Significant. rSO2: regional tissue oximetry; *Lac* blood lactate, *CPK* creatine phosphokinase, *LDH* lactic dehydrogenase, *AST* aspartate aminotransferase, *ALT* alanine aminotransferase

**Fig. 3:**
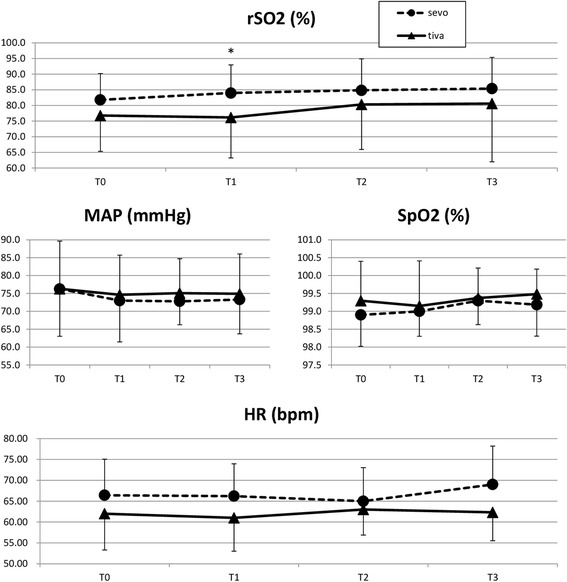
Postoperative data. All data presented as mean (standard deviation). * Significant. rSO2: regional tissue oximetry. MAP: mean arterial pressure. SpO2: saturation of peripheral oxygen. HR: heart rate. Bpm: beats per minute. T0: end of surgery, T1 4 h after surgery, T2: 12 h after surgery, T3: 20 h after surgery

There were no significant differences between groups in the recorded postoperative hemodynamic parameters (Fig. 3), nor in transfusion incidence, surgical site infection incidence, duration of surgery and surgical re-exploration incidence (Table 1).

## Discussion

In our study, we tried to assess the damage from ischemia reperfusion in two groups of patients undergoing DIEP flap for breast reconstruction.

As a marker of ischemia reperfusion, regional tissue O2 saturation in the 24 h following surgery was evaluated, along with blood levels of lactate, transaminases, lactic dehydrogenase and creatine phosphokinase. Conversely, the failure of the microsurgical flap was not considered a valid outcome to assess the damage from ischemia reperfusion since it is influenced by numerous technical variables (proper surgical technique, involvement of more than one microvascular surgeon, intraoperative fluid administration). However, the incidence of surgical site infection and surgical re-exploration were recorded.

The regional tissue oximetry is based on blood chromophores’ different characteristics of absorption of the near infrared light, in particular hemoglobin and deoxyhemoglobin’s absorption. This system, reflecting the balance between oxygen supply and demand, is a validated non-invasive and reproducible [12] method for continuous surveillance of oxygenation and perfusion of the analyzed tissue. In plastic surgery this technology is already widely used to monitor free tissue transfers [13, 14], not only for breast free flaps, but also in head, neck [15, 16] and lower extremity flaps [17].

Our results show that there is a significant increase of the average value of rSO2 measured according to INVOS at 4 h after surgery in the BAL group. It was also increased in other time frames and compared to average values, but not in a statistically significant manner. The CPK at the end of the surgery is also significantly lower in the sevoflurane group, but the difference in the two groups tends to disappear in later times.

From the results obtained, we can assert that there is an improvement of flap oxygenation with sevoflurane preconditioning, but this effect seems to be limited in time, and as hours pass the level of tissue oxygenation returns comparable to the one of not preconditioned tissue.

Congruent with our results, Balzan and colleagues [18] in their study on animals subjected to hepatic preconditioning with sevoflurane, found lower levels of C-reactive protein after ischemia, which increased in following time frames, and they concluded that the protective effect is mainly circumscribed to the initial phase of injury whereas it lessens as time elapses.

In a recent review, Swyers [19] described two possible phases of sevoflurane protection in ischemia reperfusion injury, with a mechanism similar to the protective effect of ischemic preconditioning. Swyers attributed the protective effect of volatile anesthetics to their ability to interact with the multi-signaling pathway of mitochondrial K-ATP channels. The action mechanism proposed was to hinder the entry of calcium ions in the mitochondria, allowing the functionality of the mitochondrial membrane to remain unaltered, mimicking the early phase of ischemic preconditioning. Most of studies in literature have focused on the early phase of protection, but the duration of the beneficial effects of preconditioning with volatile anesthetics remains uncertain. The early phase protection appears immediately after ischemic stimuli, but disappears within 2–4 h. The late phase protection emerges around 12 to 24 h after the initial ischemic preconditioning and lasts for 2 or 3 days [20, 21]. Some studies [22, 23] have identified adenosine and nitric oxide as the vasoactive mediators that begin a complex signal transduction cascade involving the activation of transcription factors which results in the synthesis of effector proteins. These effector proteins may confer cytoprotection in the second phase of prolonged ischemic stress. Our results may indicate a slight decrease in the protective action of sevoflurane during this phase of late protection.

The main factors that could explain our dissonant results compared to current literature are the administration of sevoflurane and the examined population.

Isofluorano studies have shown a dose-dependent protective effect of anesthetic preconditioning, reveling that higher concentrations of volatile anesthetic are more protective than lower concentrations [24–26]. On the other hand, many studies show benefits of preconditioning only after prolonged pre ischemic administration of sevoflurane [27, 28]. It is possible that a protective effect would have occurred if we had planned higher concentrations or a longer administration of sevoflurane in our study.

Further considerations regard the female population under examination. Many studies on animals and on humans have reported that female hearts have higher tolerance for ischemic injury compared to males’ [29, 30]. This difference seems to be determined by the effects of female hormones. Estrogen contributes to a natural cardioprotection in adult women by reducing oxidative stress, acting on inflammation factors and improving coronary flow [31, 32].

In his study on rabbits, Wang [33] speculates that isoflurane’s late phase protection is only active for males, not for females. This gender specificity would be due to the high baseline levels of endothelial nitric oxide synthase (eNOS) expressed in females, due to estrogen, which naturally give additional protection against myocardial infarction, but do not grant the benefit of preconditioning effects of volatile anesthetics in females, through a mechanism that has not yet completely been understood.

Another animal study [34] indicated that anesthetic postconditioning could also lead to gender differences in cardioprotection. In this case, the protective effect on males is expressed through the key role of the phosphatidylinositol-3-kinase (PI3K / Akt) pathway. Due to estrogen influence, in female rats the PI3K/Akt pathway was already markedly overexpressed and was not activated any further by postconditioning with sevoflurane.

Sevoflurane preconditioning has been widely investigated in the context of cardiac surgery and clinical studies have shown that sevoflurane is cardio-protective in cardiac surgery patients [35], but this effect is ambiguous on other tissues and organs in different surgical fields [6]. Some studies on patients undergoing liver resection [36, 37] did not show significant differences in hepatocyte and endothelial dysfunction markers, nor in postoperative outcomes between the groups. In addition, results on kidney function are controversial, and they were unable to report definitive improvements in the degree of kidney injury [38, 39].

About microvascular breast flap surgery, there are no studies that have investigated the protective effect of sevoflurane on ischemia reperfusion injury, also due to the lack of highly specific blood markers. In our case, we considered regional O2 inflow measured by NIRS technology and common markers of cellular damage valid indicators to assess the damage from IR. Although we excluded patients with vascular or bleeding pathologies from the study, the relation between tissue perfusion and management is complex and multifactorial, so we tried to minimize counfunding factors by using strict protocols. Mean arterial pressure was maintained in established limits using opioids and hypnotic drugs, fluid therapy was also used to regulated ABP and to replace loss, the temperature was also maintained constant in the operating room.

Regarding the weaknesses of our study, this is a multicentric trial, so the surgery was conducted by two different equipes, this obviously determined a margin of procedure variability. To minimize this inter-instituitonal gap we standardized the anesthesiological management while the surgical procedure was discussed and decided out of accorded with the two operative teams. Other limits: the number of patients was relatively small and we used a single preconditioning protocol; our study may represent a starting point for future studies, which will also consider different volatile anesthetic concentrations and different preconditioning timings.

## Conclusions

In conclusion, the results regarding the rSO2 values recorded indicate an initial protective effect of sevoflurane in the treated group, which does not persist in the long-term. We believe that a prospective, randomized, placebo-controlled trial that clarifies the effects of volatile anesthetics preconditioning with a larger number of patients could lead in the future to the creation of an anesthesia plan not only based on the type of surgery, but even “patient tailored”.

## Abbreviations

ABP: Arterial blood pressure
ALT: Alanine aminotransferase
ASA: American society of anaesthesiologists
AST: Aspartate aminotransferase
ATP: Adenosine triphosphate
BAL: Balanced anesthetic technique
BMI: Body mass index
CPK: Creatine phosphokinase
DIEP: Deep inferior epigastric perforator
eNOS: Endothelial nitric oxide synthase
etCO2: End-tidal CO2
HR: Heart rate
INVOS™: In-vivo optical spectroscopy system
IR: Ischemia-reperfusion
LDH: Lactic dehydrogenase
NIRS: Near-infrared spectroscopy
PI3K/Akt: Phosphatidylinositol-3-kinase
rSO2: Regional tissue oximetry
SpO2: Pulse oximetry
TCI: Target-controlled infusion
